# Personalized Decision-Making in Risk-Reducing Surgery of the Ovaries

**DOI:** 10.1001/jamanetworkopen.2026.3404

**Published:** 2026-03-31

**Authors:** Mary B. Daly, Brian L. Egleston, Kaitlyn Lew, Lisa Bealin, Alexander Husband, Jill E. Stopfer, Pawel Przybysz, Olga Tchuvatkina, Yu-Ning Wong, Judy E. Garber, Timothy R. Rebbeck

**Affiliations:** 1 Department of Clinical Genetics, Fox Chase Cancer Center, Temple University Health System, Philadelphia, Pennsylvania; 2 Department of Biostatistics, Fox Chase Cancer Center, Temple University Health System, Philadelphia, Pennsylvania; 3 Division of Cancer Genetics and Prevention, Department of Medical Oncology, Dana-Farber Cancer Institute, Boston, Massachusetts; 4 Division of Hematology and Oncology, Perelman School of Medicine, University of Pennsylvania, Philadelphia; 5 Corporal Michael J. Crescenz VA Medical Center, Philadelphia, Pennsylvania

## Abstract

**Importance:**

Premenopausal women at familial or hereditary risk of ovarian cancer must weigh the risks and benefits of risk-reducing surgery. How they navigate this decision is not well understood.

**Objective:**

To assess the factors that motivate women to seek risk-reducing surgery for ovarian cancer.

**Design, Setting, and Participants:**

A discrete choice experiment enrolled premenopausal women seeking genetic testing for ovarian cancer risk at 2 tertiary-level cancer centers between August 2019 and January 2022. The experiment was conducted twice, once just prior to pretest counseling and again approximately 2 months later, after receiving results of genetic testing. Data analysis was conducted between June 2022 through January 2026.

**Main Outcomes and Measures:**

Conjoint analysis to assess factors influencing cancer-prevention decisions in women with risk of familial and hereditary ovarian cancer. Conjoint analysis was used to investigate which attributes were the most salient for women when considering treatment scenarios, with demographic and clinical data as modifiers. Participants were asked to choose between sets of randomly permuted scenarios specifying type of treatment, risk and age of ovarian cancer, risk of heart disease and osteoporosis, and features of menopause.

**Results:**

The participants included 355 women (median age, 37 years [range, 21-55 years]): 10.4% were Black, 7.6% were Hispanic, 85.1% were White, and 79.2% were college graduates. Family history of breast cancer was reported by 45.1%, ovarian cancer by 13.0%, and 11.3% had a personal history of breast cancer. Women preferred risk-reducing salpingo-oophorectomy (RRSO) to risk-reducing salpingectomy (RRS) or observation (odds ratio [OR], 1.24; 95% CI, 1.10-1.39). Differences in osteoporosis (OR, 0.83; 95% CI, 0.78-0.89) and heart disease (OR, 0.78; 95% CI, 0.73-0.84) risks affected choices. Preferences favored natural menopause (OR, 1.21; 95% CI, 1.11-1.33) and milder menopausal symptoms (OR, 0.66; 95% CI, 0.61-0.70). Family history of ovarian cancer increased preference for RRSO (OR, 1.26; 95% CI, 1.05-1.52), with a reduced effect in older women. Personal breast cancer history, ancestry, and genetic test results were not significant factors.

**Conclusions and Relevance:**

This survey study using conjoint analysis highlights the ability of women to weigh competing risks in reducing ovarian cancer. Counseling models that address decision complexity and incorporate personalized guidance are likely to improve the decision-making process.

## Key Points

**Question:**

What are the factors that women consider when making complex decisions regarding risk-reducing surgery for ovarian cancer risk?

**Findings:**

This survey study in 355 premenopausal women with familial or hereditary risk of ovarian cancer found that participants significantly preferred risk-reducing salpingo-oophorectomy overall. Increased risk of osteoporosis and heart disease reduced this preference.

**Meaning:**

Results of this study suggest that counseling models that provide reliable information about the surgery and its sequelae may improve the decision-making process.

## Teaser

This survey study assesses factors that women at familial or hereditary risk of ovarian cancer consider with regard to risk-reducing surgery.

## Introduction

The discovery of the breast cancer 1 and 2 (*BRCA1/2*) genes in the 1990s accelerated our understanding of genetic risks for breast cancer, high-grade serous ovarian cancer, and other cancers. Women who have inherited a *BRCA1/2* pathogenic variant (PV) have a 10% to 45% lifetime risk of ovarian cancer, which is 10 to 30 times higher than the general population.^1,2,3^ PVs in these and other genes have been associated with an increased risk of ovarian cancer and are routinely included in multigene panels for hereditary risk testing.^4,5^

Women with a familial or hereditary risk of ovarian cancer face difficult decisions about cancer screening and risk-reducing choices. Risk-reducing salpingo-oophorectomy (RRSO), the surgical removal of the ovaries and fallopian tubes, is associated with an 80% reduction in the risk of ovarian, fallopian tube, and peritoneal cancer and has been established to offer the best protection from ovarian cancer.^6,7,8^ Recently, the realization that most ovarian cancer originates in the fimbriated end of the fallopian tube, not in the ovary,^9^ has led to risk-reducing salpingectomy (RRS), which is removal of the fallopian tubes alone before menopause, with delayed removal of the ovaries after menopause. RRS represents an emerging alternative strategy for women who are premenopausal at high risk and wish to avoid early menopause. Although RRS is not the standard of care due to limited data regarding its safety and efficacy, women are becoming aware of this procedure and its uptake is increasing.^10^ The nonsurgical option is close surveillance, which, in the absence of effective screening, monitors for the onset of clinical symptoms that often detect the disease at an advanced stage.

The decision to undergo RRSO or RRS is complex^11^ due to the variability in individual ovarian cancer risk, its timing, and potential adverse events from the surgical procedure itself.^12^ Risk estimates can differ based on the specific PV and its specific gene site.^13,14,15^ Early surgical menopause brings immediate menopausal symptoms, fertility loss, and long-term risks, including increased cardiovascular disease incidence and mortality, osteoporosis, and bone fractures.^16,17,18,19^ As a result of these complexities, the decision to undergo risk-reducing surgery varies among women and can be influenced by factors such as age, individual ovarian cancer risk, comorbidities, personal and family history, and stage of life.^20,21^

Risk assessment and counseling are used to assist women at high risk choosing prevention strategies^22^; there is limited information about the factors that affect cancer-prevention decision-making in women with a familial or hereditary risk of ovarian cancer, or the efficacy of decision aids to assist in choosing management strategies.^23^ Women may have different preferences based on demographic, personal, familial, and clinical factors. This multisite study aimed to assess the factors associated with cancer-prevention decisions in women with familial and hereditary ovarian cancer risk. We examined pretest and posttest risk and health state risk tolerances and their modifiers that could affect the choice of RRSO, RRS, or surveillance.

## Methods

### Study Participants

For this survey study, written informed consent was obtained from 355 women who were premenopausal from the Risk Assessment Program at Fox Chase Cancer Center and the Center for Cancer Genetics and Prevention at the Dana-Farber Cancer Institute between August 2019 and January 2022. Data analysis was performed between June 2022 through January 2026. Both institutions provide comprehensive genetic services, including testing for PVs in ovarian cancer genes, including *BRCA1, BRCA2, BRIP1, RAD51, RAD51B, RAD51C, RAD51D, PALB2, MLH1, MSH2, MSH6,* and *EPCAM*. PVs in these genes are found in approximately 10% of women who undergo testing for hereditary breast/ovarian cancer risk. Participants had a personal history of breast cancer and/or a familial risk of hereditary breast or ovarian cancer and sought genetic counseling and testing. Participants completed the study survey twice, first at the completion of the pretest counseling and again after the posttest session when genetic test results are disclosed to assess the potential role of the test results in preferences. This study was reviewed and approved by the Dana-Farber Cancer Institute’s and Fox Chase Cancer Center’s institutional review boards. Data have been anonymized.

### Recruitment

Eligibility criteria for the study included women aged 18 years or older with at least 1 intact ovary, English-speaking, and seeking genetic evaluation for hereditary breast/ovarian cancer risk. Women were identified through intake data and approached by the project managers after their initial counseling. Eligible women who agreed to participate were given access to a password-protected survey site. There was a 10.0% loss of participants who either did not start the survey or did not complete the survey (Figure).

**Figure.**
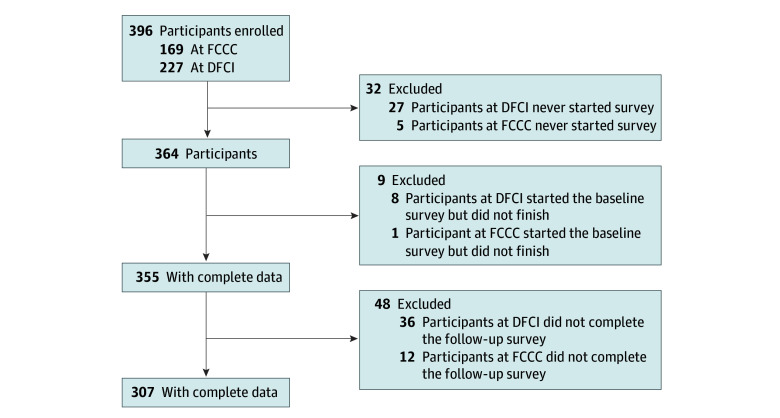
Flow Diagram of the Study Schema DFCI indicates Dana-Farber Cancer Institute; FCCC, Fox Chase Cancer Center.

### Survey Tool

We designed a discrete choice experiment using a conjoint analysis instrument to assess which risks are most relevant to women considering the option of RRSO, RRS, or surveillance to manage their ovarian cancer risk. This instrument was designed according to American Association for Public Opinion Research (AAPOR) reporting guideline for the design, data collection, and analysis of a quality survey. Conjoint analysis was originally developed to measure the association between product attributes and consumer choice (eg, price, accessories).^24^ In the medical setting, conjoint analysis can assess the association of multiple factors with patients’ health-related decision-making. This methodology assumes that decisions can be described by the attributes and the values that a patient places on those attributes, depending on their perceived relative strength.^25,26,27^ The attributes we chose were (1) type of risk management strategy, (2) estimated lifetime risk of ovarian cancer, (3) estimated age at diagnosis of ovarian cancer, (4) estimated risk of developing osteoporosis after surgery, (5) estimated risk of developing heart disease after surgery, (6) timing of menopausal symptoms (after surgery vs natural age), (7) and the estimated severity of menopausal symptoms after surgery. Each attribute is assigned a level using estimates from published data^17,18,28^ (eTable 1 in Supplement 1). In each scenario, participants chose between 2 combinations of attribute levels (eTable 2 in Supplement 1). We used the balanced overlap design, an experimental design that balances the amount of overlap that occurs in the choice sets, as implemented in Lighthouse Studio software version 9.8.1 (Sawtooth Software) to permute attributes shown to participants.

The survey tool is composed of 5 parts. In part 1, participants were asked to provide demographic and clinical data, including age, education, race or ethnicity, childbearing status, both family and personal history of cancer, family history of cardiovascular disease, and family history of osteoporosis as they are potential modifiers of the outcome. Part 2 provided basic information about the possible treatments that can be considered to reduce ovarian cancer risk, such as RRSO, RRS, and surveillance. Part 3 included basic information about potential risks of postsurgical menopause, cardiovascular disease, and osteoporosis. Potential management strategies of these risks were not included. Part 4 provided some basic descriptions of cancer statistics terminology. Part 5 presented the clinical scenarios and asked participants to choose between a pair of hypothetical scenarios based on the level of the attributes presented. This choice experiment was repeated 5 times.

### Statistical Analysis

The conjoint analysis design assesses tradeoffs so that we can identify attribute combinations that maximize participant utility. We characterized the data using descriptive statistics and evaluated differences by lost to follow-up status using *t* tests and Fisher exact tests. Conditional logistic regressions assessed the association of attributes with choices.^29^ We used Wald tests on coefficients^30^ and accounted for the repeated within-participant design via cluster-corrected standard errors. We used interaction terms to compare precounseling with postcounseling responses. Interactions also assessed whether demographic characteristics moderated attribute effects on choices. Variables included in the interaction analysis included age, self-reported parity, education, positive gene result, family history of ovarian cancer (self-report), personal or family history of breast cancer (self-report), other cancer (self-report), and self-reported family history of heart attack, coronary artery disease, high cholesterol, congestive heart failure, and bone fracture. To further investigate treatment strategy in terms of moderating factors, we used 3 logistic regressions estimated by generalized estimating equations and incorporating bootstrap SEs to compare attributes associated with choosing between scenarios in which pairwise risk-reducing treatment strategies differed. A 2-sided *P* < .05 was used to represent statistical significance. Data were analyzed using Stata version 18 (StataCorp).

## Results

A total of 396 women were enrolled in this study, 32 did not start the survey, 9 started but did not finish the survey, and 355 women completed the baseline survey (Figure). The median age was 37 years (range, 21-55 years). Among demographic characteristics, 10.4% reported being Black, 7.6% reported being Hispanic, and 85.1% reported being White; 79.2% were college graduates; and 56.6% of the women had children (Table 1). In addition, 11.3% had a personal history of breast cancer, 45.1% had a family history of breast cancer, and 13.0% had a family history of ovarian cancer (Table 1). Forty-seven participants (13.0%) had a positive test result. Forty-eight participants (14.0%) did not complete the follow-up survey. Those who did not complete the follow-up survey were more likely to be Asian (13.0% not completing vs 1.0% completing; *P* = .001) and more likely to be from the Dana-Farber Cancer Institute (75.0% not completing vs 51.0% completing). There were no other differences by follow-up status.

**Table 1.** Description of the Study Sample (N = 355)

Variable	No. (%)
Age (n = 27 missing), y	
Mean (SD)	37.11 (8.98)
Median (IQR) [range]	36.0 (30.0-44.0) [21.0-55.0]
Year	
2019	28 (7.9)
2020	97 (27.3)
2021	226 (63.7)
2022	4 (1.1)
Family history of ovarian cancer (self-report)	46 (13.0)
History of breast cancer (self-report)	
Personal	40 (11.3)
Family	160 (45.1)
Family history of following (self-report)	
Coronary artery disease	27 (7.6)
Heart attack	58 (16.3)
Congestive heart failure	27 (7.6)
High cholesterol	153 (43.1)
Osteoporosis (loss of bone strength)	60 (16.9)
Bone fracture due to osteoporosis	9 (2.5)
Race and ethnicity^a^	
American Indian or Alaskan Native	7 (2.0)
Asian	10 (2.8)
Black	37 (10.4)
Hispanic or Latino	27 (7.6)
Other race^b^	13 (3.7)
White	302 (85.1)
Do you have children?	
Yes	201 (56.6)
Educational level	
High school or less	24 (6.8)
Some college	50 (14.1)
College graduate	281 (79.2)
Site	
Fox Chase Cancer Center	163 (45.9)
Dana-Farber Cancer Institute	192 (54.1)

^a^ Race and ethnicity were self-reported.

^b^ Other race was self-reported free text of one’s race. The responses were nonspecific and no further breakdown of this classification is available.

Table 2 presents the association of attributes with choices. Responses from pretest and posttest counseling surveys were combined since we did not find any statistically significant interactions by counseling period. Women preferred RRSO overall (odds ratio [OR], 1.24; 95% CI, 1.10-1.39) compared with RRS. RRS was the least preferred treatment, although the finding for surveillance and RRS was not statistically significant (OR, 1.12; 95% CI, 0.98-1.28). The odds of choosing a choice set decreased as the difference in ovarian cancer risk between choice sets increased (per 10% increase: OR, 0.66; 95% CI, 0.62-0.71; for example, OR = 0.44 for a 20% increase). Women preferred choice sets in which the age of onset was delayed (OR, 1.03; 95% CI, 1.02-1.04). Increased risk of osteoporosis and heart disease also reduced preferences for choice sets, but not as much as the risk of ovarian cancer (OR, 0.83 for 10% increase; 95% CI, 0.78-0.89 for osteoporosis; and OR, 0.78 for 10% increase; 95% CI, 0.73-0.84 for heart disease). Women preferred scenarios with a natural age of menopause (OR, 1.21; 95% CI, 1.11-1.31) and reduced severity of menopausal symptoms (OR, 0.66 for 1 level worsening of symptoms; 95% CI, 0.61-0.70).

**Table 2.** Association of Attributes With Choices as Differences Between 2 Choices

Attribute	Odds ratio (95% CI)	*P* value
Treatment		
RRS	1 [Reference]	NA
Surveillance	1.12 (0.98-1.28)	.10
RRSO	1.24 (1.10-1.39)	<.001
Risk of ovarian cancer (10% increase in difference)	0.66 (0.62-0.71)	<.001
Age that cancer occurs	1.03 (1.02-1.04)	<.001
Risk of osteoporosis (10% increase)	0.83 (0.78-0.89)	<.001
Risk of heart disease (10% increase)	0.78 (0.73-0.84)	<.001
Timing of menopause		
Immediate menopause after treatment	1 [Reference]	NA
Natural age of menopause	1.21 (1.11-1.31)	<.001
Quality of menopausal symptoms (per severity increase)	0.66 (0.61-0.70)	<.001

Abbreviations: NA, not applicable; RRS, risk-reducing salpingectomy; RRSO, risk-reducing salpingo-oophorectomy.

The conjoint analysis utilities associated with each attribute can be estimated by taking the log of the ORs. Our findings suggest that the risk of ovarian cancer (utility = log[0.66] = −0.42) and the quality of menopausal symptoms (utility = log[0.66] = −0.42) were the most salient concerns. While important to decision-making, the type of treatment, risks of osteoporosis and heart disease, and timing of menopause are less influential based on the magnitude of utilities.

In eTable 3 in Supplement 1, we compared the association of attributes with choices in pairwise comparisons of the prevention strategies. For example, we compared attributes affecting the choice of RRSO (binary response = 1) vs surveillance (binary response = 0 as the reference). General inferences about the effect of attributes did not change much in this subanalysis, although the importance of heart disease postconsultation on choice increased when surveillance was an option. The lack of other substantial differences suggests that the type of surgery by itself is not a major factor in decision-making (eg, there is not strong evidence that surveillance is inherently preferred over surgery, all else being equal). Rather, it is the risk profile of the choice set rather than type of surgery that is a main motivator of decision-making.

The finding that higher ovarian cancer risk is less likely to be associated with choosing a surgical scenario is attenuated by an OR of 1.01 for each year a woman gets older (95% CI, 1.00-1.01), suggesting that ovarian cancer risk concerns are more salient for younger women. Women with children are more likely to choose more aggressive surgery over surveillance. Those with a higher educational level tended to prefer surveillance over RRS, but education did not moderate the preference for RRSO over surveillance. Table 3 presents statistically significant interactions between demographic and medical history factors and scenario attributes in the time period combined data. Family history of ovarian cancer increases preference for RRSO (OR, 1.26; 95% CI, 1.05-1.52) with a reduced effect for older women. Having a family history of ovarian cancer attenuated the relationship of osteoporosis risk with choice, suggesting that the osteoporosis risk is less important for those who might perceive themselves as being at higher risk of ovarian cancer. A family history of breast cancer lessened the importance of worsening menopausal symptoms on choice of treatment, suggesting that the severity of menopausal symptoms is less important to those who consider themselves at familial risk. Having a family history of a heart disease risk factor (eg, coronary artery disease, congestive heart failure) affected choices. A family history of coronary artery disease strengthened preferences for lower heart disease risk scenarios, while a family history of congestive heart failure lessened the importance of risk of osteoporosis on choices. A family history of bone fracture related to osteoporosis increased the preference for a natural age at menopause. Factors such as personal history of breast cancer and family history of cancer were not significantly related to scenario choice. A positive ovarian cancer–related genetic test result, seen in 13% of the study population, did not moderate preferences. The interaction OR for the moderation of a positive genetic test with the risk of ovarian cancer is 1.01 (95% CI, 0.84-1.21), with risk of heart disease is 0.95 (95% CI, 0.80-1.14), and with quality of menopausal symptoms is 0.97 (95% CI, 0.79-1.20).

**Table 3.** Interaction Effect Estimates Comparing Factors That Moderate RRS or RRSO Relative to Surveillance

Interaction	Interaction term estimate	
	Odds ratio (95% CI)	*P* value
Age with risk of ovarian cancer	1.01 (1.00-1.01)	.04
Children with RRS over surveillance	1.41 (1.08-1.85)	.01
Children with RRSO over surveillance	1.29 (1.02-1.63)	.03
Higher education with risk of ovarian cancer	0.87 (0.79-0.95)	.002
Higher education with surveillance over RRS	1.27 (1.02-1.62)	.05
Family history of ovarian cancer (self-report) with risk of osteoporosis	1.26 (1.05-1.52)	.01
Family history of breast cancer (self-report) with quality of menopausal symptoms	1.20 (1.05-1.37)	.01
Family history of coronary artery disease with risk of heart disease	0.73 (0.53-1.00)	.05
Family history of high cholesterol with RRSO over surveillance	1.28 (1.01-1.61)	.04
Family history of CHF with risk of osteoporosis	1.27 (1.01-1.60)	.04
Family history of bone fracture with natural age at menopause	1.27 (1.04-1.56)	.02

Abbreviations: CHF, congestive heart failure; RRS, risk-reducing salpingectomy; RRSO, risk-reducing salpingo-oophorectomy.

## Discussion

The introduction of clinical genetic testing for hereditary cancer risk into the clinical setting has led to new strategies for cancer risk reduction, especially for cancers that lack effective screening options. Our work in the setting of familial and hereditary ovarian cancer risk sheds light on how women at increased risk of ovarian cancer navigate the complex decision of choosing risk-reducing strategies. A conjoint analysis allows for the simultaneous assessment of various risk aspects in this population of women and helps identify the most important factors in decision-making, particularly in genetic or familial risk contexts, where decisions affect both the individual and family.

Here we report the key factors associated with women’s decisions in ovarian cancer risk management. It is possible that a general preference for or against surgical intervention could impact decisions. While the type of strategy did not play a substantial role, the degree to which a strategy reduced ovarian cancer risk or impacted the quality of menopausal symptom severity seemed to be more important to women. Our findings are consistent with studies that have found that uncertainty about risk-reducing surgery for ovarian cancer risk protection limits its acceptance.^31,32^ While scenarios with higher risks of osteoporosis, heart disease, and worsening menopausal symptoms lessened the choice of those attribute sets, these had less impact on choice than cancer risk.

The interaction (ie, moderator or effect modifier) analyses indicate how personal and familial experiences may affect women’s decision-making process. Women may downplay the severity of adverse effects of surgery if they have witnessed cancers in their families and are more willing to endure those adverse effects to avoid developing the same cancer that they have observed. In addition to family history, women with children preferred more aggressive treatment, perhaps with the hope that doing so would allow them to have a longer lifespan over which they can be part of their children’s lives. This may reflect the fact that women with children are more likely to have completed their families and are therefore less likely to avoid RRSO. There was no significant shift in preferences in the posttest survey among women who tested positive for an ovarian cancer–related PV. This is a population of women who are already alerted to their cancer risks and in whom the test result may be only 1 factor explaining their potential risk. Their preferences may be also directed by relevant personal exposure to familial ovarian cancer.

Our findings provide insights that may inform how counselors and clinicians present the range of risks and benefits associated with ovarian cancer risk reduction. While our data support the central role of the magnitude of ovarian cancer risk as a motive for choice of treatment, they also suggest that women at increased risk of ovarian cancer consider a range of additional factors when assessing preventive strategies for ovarian cancer. Almost all our attributes affected choices, but the magnitudes of coefficients suggest that some attributes had stronger effects than others. The ORs for the risk of ovarian cancer on choices have larger magnitudes than the ORs for the risk of heart disease or osteoporosis. The preference for a natural age of menopause was less pronounced than the desire to avoid severe menopausal symptoms, suggesting that symptoms are a greater concern than age at menopause. Additionally, family history of cancer and long-term conditions linked to premature menopause, such as cardiovascular disease and osteoporosis, were factors in women’s decisions.

As genetic counseling resources are strained,^33^ a better understanding of the factors influencing women’s choices for ovarian cancer risk reduction can help personalize and streamline risk messages.^34^ In addition to discussing ovarian cancer risk, it is important to provide information on a woman’s personal and family history of cardiovascular disease and osteoporosis. Future research could further investigate how concerns about fertility preservation, sexual functioning, and options for medical management of menopausal symptoms affect decisions.^35^

### Limitations

This study has limitations. This discrete choice experiment presents artificial scenarios, so they can only help us understand how women might manage a theoretical risk of ovarian cancer. Participants were primarily White, well educated, and recruited from academic cancer centers, which may not reflect the broader community where health literacy and numeracy (a measure of confidence to use basic math in real-life situations) vary. Additionally, pretest and posttest counseling was not monitored for consistency, and the counseling content may have affected their responses. Despite these limitations, this study has many strengths. It uses conjoint analysis, which allows investigators to assess how patients make trade-offs in complex decisions such as this. The tool is web-based suggesting that this kind of instrument could be useful for personalizing individual patient health preferences.

## Conclusions

Results of this study suggest that conjoint analysis is a promising tool to assess complex risk management decisions in the setting of hereditary ovarian cancer. Women with a hereditary risk of ovarian cancer are recommended to undergo risk-reducing surgery for primary prevention. The decision-making process for premenopausal women is fraught with multiple competing health risks associated with the immediate and long-term loss of ovarian hormones. In many cases, these associated concerns may not be a standard component of the counseling model. This conjoint analysis experiment illustrated the ability of women to incorporate the level of these competing risks in the setting of the benefit of reduced ovarian cancer risk as they considered their options. Counseling models that recognize the complexity of ovarian cancer risk reduction and provide personalized guidance tailored to each woman’s unique needs are likely to improve the decision-making process and enhance patient outcomes.

## Notes

Supplement 1. **eTable 1.** Attributes Table for RRS, RRSO, and Surveillance **eTable 2.** Example 1 and Example 2 **eTable 3.** Associations With Choosing Scenarios in Which the Prevention Strategy Differed

Supplement 2.
Data Sharing Statement
