# Supplementary material for: Personalized Decision-Making in Risk-Reducing Surgery of the Ovaries
Source: JAMA Netw Open. 2026 Mar 31;9(3):e263404. doi: 10.1001/jamanetworkopen.2026.3404 (PMC13040396; doi:10.1001/jamanetworkopen.2026.3404)
Supplement: Supplement 1. — eTable 1. Attributes Table for RRS, RRSO, and Surveillance eTable 2. Example 1 and Example 2 eTable 3. Associations With Choosing Scenarios in Which the Prevention Strategy Differed [file jamanetwopen-e263404-s001.pdf]

Supplemental Online Content

Daly MB, Egleston BL, Lew K, et al. Personalized decision-making in risk-reducing surgery of the ovaries. *JAMA Netw Open*. 2026;9(3):e263404. doi:10.1001/jamanetworkopen.2026.3404

eTable 1. Attributes Table for RRS, RRSO, and Surveillance
eTable 2. Example 1 and Example 2
eTable 3. Associations with choosing scenarios in which the prevention strategy differed

This supplemental material has been provided by the authors to give readers additional information about their work.

© 2026 Daly MB et al. *JAMA Network Open*.

eTable 1: Attributes Table for RRS, RRSO, and Surveillance

Attribute	Level	Value
**Lifetime risk of ovarian cancer**	1	10%
	2	20%
	3	40%
**Probable age of onset of ovarian cancer**	1	30 yr.
	2	40 yr.
	3	50yr.
**Lifetime risk of osteoporosis**	1	10%
	2	20%
	3	30%
**Lifetime risk of CVD**	1	10%
	2	20%
	3	40%
**Type of treatment**	1	Surveillance
	2	Risk-reducing salpingectomy
	3	Risk reducing salpingo-oophorectomy
**Timing of menopausal Symptoms**	1	Immediately after surgery
	2	Natural age
**Severity of menopausal symptoms**	1	No Symptoms
	2	Moderate symptoms
	3	Severe symptoms

© 2026 Daly MB et al. *JAMA Network Open*.

eTable 2: Example 1

Risk of ovarian Cancer	35 per 100 (35%)	20 per 100 (20%)
Age if cancer develops	65 years	55 years
Risk of osteoporosis	30 per 100 (30%)	10 per 100 (10%)
Risk of heart disease	20 per 100 (20%)	5 per 100 (5%)
Type of Treatment	Surgery to remove your ovaries and Fallopian tubes (RRSO)	Surveillance
Timing (Age) of menopausal symptoms	Immediately after treatment	Natural age
Quality of menopausal symptoms	Mild	Severe

© 2026 Daly MB et al. *JAMA Network Open*.

eTable 2: Example 2

Risk of ovarian cancer	35 per 100 (35%)	15 per 100 (15%)
Age if cancer develops	60 years	50 years
Risk of osteoporosis	15 per 100 (15%)	20 per 100 (20%)
Risk of heart disease	15 per 100 (15%)	30 per 100 (30%)
Type of treatment	Surgery to remove your ovaries and fallopian tubes (RRSO)	Surgery to remove your ovaries and fallopian tubes (RRSO)
Timing (Age) of menopausal symptoms	Natural age	Immediately after treatment
Quality of menopausal symptoms	Severe	Moderate

© 2026 Daly MB et al. *JAMA Network Open*.

eTable 3. Associations with choosing scenarios in which the prevention strategy differed

	3A. RRSO versus Surveillance (reference)		3B. RRSO versus RRS (reference)		3C. RRS versus Surveillance (reference)	
	Odds Ratio (95% CI)	P-value	Odds Ratio (95% CI)	P-value	Odds Ratio (95% CI)	P-value
**Collapsed time points**						
Risk of ovarian cancer (per 10% reduction with RRSO)	0.63 (0.57, 0.69)	<0.001	0.68 (0.62, 0.76)	<0.001	0.62 (0.57, 0.68)	<0.001
Age that cancer occurs	1.03 (1.01, 1.04)	0.002	1.04 (1.02, 1.05)	<0.001	1.04 (1.02, 1.06)	<0.001
Risk of osteoporosis (per 10% increase with RRSO)	0.82 (0.73, 0.92)	0.001	0.80 (0.71, 0.91)	0.001	0.83 (0.73, 0.93)	0.002
Risk of heart disease (per 10% increase with RRSO)	0.79 (0.70, 0.88)	<0.001	0.80 (0.71, 0.9)	<0.001	0.72 (0.64, 0.81)	<0.001
Timing of menopause						
Immediate menopause after treatment	Reference		Reference		Reference	
Natural age of menopause with RRSO choice	1.43 (1.24, 1.64)	<0.001	1.10 (0.94, 1.29)	0.217	1.15 (0.99, 1.33)	0.066
Worsening of menopausal symptoms with RRSO choice	0.68 (0.61, 0.75)	<0.001	0.60 (0.54, 0.67)	<0.001	0.70 (0.62, 0.78)	<0.001
**Risk of heart disease by survey wave**	**p=0.031 for interaction**		**P>0.05 for interaction**		**p=0.011 for interaction**	
Risk of heart disease effect pre-counseling	0.88 (0.76, 1.01)	0.074			0.83 (0.72, 0.96)	0.010
Risk of heart disease effect post-counseling	0.69 (0.59, 0.81)	<0.001			0.62 (0.52, 0.74)	<0.001

Supplemental description of Supplemental Table 3. In Supplemental Table 3, we present attributes that are associated with the 3-way pairwise comparisons of treatments. In each table, we compared the less extensive treatment modality to the most extensive treatment modality (i.e., surveillance is least extensive, and risk-reducing salpingo-oophorectomy is most extensive). We found pre- to post-test changes in preferences only for lower risk of heart disease scenarios when comparing risk-reducing salpingo-oophorectomy or risk-reducing salpingectomy to surveillance. We report salient pre- versus post-test associations of risk of heart disease with choice separately by survey wave (Supplemental Tables 3a-3c). As there were no significant pre- to post-test survey interactions of the other variables, we present results for the combined time periods (Supplemental Tables 3a-3c).

Women preferred treatment options with the most favorable risk reduction and side effect profile. Women were less likely to choose surgical treatments with smaller benefits. The odds ratio for natural menopause age effect was larger when choosing between risk-reducing salpingo-oophorectomy to risk-reducing salpingectomy than between risk-reducing salpingo-oophorectomy versus surveillance (Supplemental Table 3a), as compared to the odds ratio for choosing between risk-reducing salpingectomy versus surveillance (p=0.041 for interaction) or choosing between risk-reducing salpingo-oophorectomy versus risk-reducing salpingectomy (p=0.014 for interaction) (Supplemental Tables 3b and 3c). When choosing between scenarios with surgical strategies versus surveillance, the impact of heart disease risk on choice strengthened pre- to post-test counseling. This suggests that the counseling session increased the salience of heart disease side effects on choices. The other side effects of strategies did not have statistically significant change pre- to post-test counseling.

© 2026 Daly MB et al. *JAMA Network Open*.
