# Supplementary material for: Personalized Decision-Making in Risk-Reducing Surgery of the Ovaries
Source: JAMA Netw Open. 2026 Mar 31;9(3):e263404. doi: 10.1001/jamanetworkopen.2026.3404 (PMC13040396; doi:10.1001/jamanetworkopen.2026.3404)
Supplement: Supplement 2. — Data Sharing Statement [file jamanetwopen-e263404-s002.pdf]

Data Sharing Statement

Daly. Personalized Decision-Making in Risk-Reducing Surgery of the Ovaries. *JAMA Netw Open*. Published March 31, 2026. doi:10.1001/jamanetworkopen.2026.3404

Data
Data available: Yes
Data types: Other (please specify)
Additional Information: All data will be made available with completion of a data use agreement. Programming code is available upon request.
How to access data: Qualified researchers may reach out to the corresponding author to request access to the data used in this manuscript.
When available: With publication

Supporting Documents
Document types: None

Additional Information
Who can access the data: Qualified researchers may reach out to the corresponding author to request access to the data used in this manuscript.
Types of analyses: n/a
Mechanisms of data availability: With the investigators support, after the approval of a proposal
